# Suppression of HBV by Tenofovir in HBV/HIV Coinfected Patients: A Systematic Review and Meta-Analysis

**DOI:** 10.1371/journal.pone.0068152

**Published:** 2013-07-10

**Authors:** Huw Price, David Dunn, Deenan Pillay, Firouze Bani-Sadr, Theodora de Vries-Sluijs, Mamta K. Jain, Noriyoshi Kuzushita, Stefan Mauss, Marina Núñez, Reto Nüesch, Marion Peters, Thomas Reiberger, Christoph Stephan, Lionel Tan, Richard Gilson

**Affiliations:** 1 Research Department of Infection and Population Health, University College London, London, United Kingdom; 2 Clinical Trials Unit, Medical Research Council, London, United Kingdom; 3 Virology, University College London, London, United Kingdom; 4 AP-HP Hôpital Tenon, Paris, France; 5 Erasmus Medical Centre, Rotterdam, The Netherlands; 6 UT Southwestern Medical Center, Dallas, Texas, United States of America; 7 Osaka National Hospital, Osaka City, Japan; 8 Center for HIV and Hepatogastroenterology, Duesseldorf, Germany; 9 Wake Forest University Health Sciences, Winston-Salem, North Carolina, United States of America; 10 University Hospital Basel, Basel, Switzerland; 11 University of California San Francisco, San Francisco, California, United States of America; 12 Medical University of Vienna, Vienna, Austria; 13 Johann Wolfgang Goethe-Universität, Frankfurt am Main, Germany; 14 Imperial College London, London, United Kingdom; Yonsei University College of Medicine, Republic of Korea

## Abstract

**Background:**

Hepatitis B coinfection is common in HIV-positive individuals and as antiretroviral therapy has made death due to AIDS less common, hepatitis has become increasingly important. Several drugs are available to treat hepatitis B. The most potent and the one with the lowest risk of resistance appears to be tenofovir (TDF). However there are several questions that remain unanswered regarding the use of TDF, including the proportion of patients that achieves suppression of HBV viral load and over what time, whether suppression is durable and whether prior treatment with other HBV-active drugs such as lamivudine, compromises the efficacy of TDF due to possible selection of resistant HBV strains.

**Methods:**

A systematic review and meta-analysis following PRISMA guidelines and using multilevel mixed effects logistic regression, stratified by prior and/or concomitant use of lamivudine and/or emtricitabine.

**Results:**

Data was available from 23 studies including 550 HBV/HIV coinfected patients treated with TDF. Follow up was for up to seven years but to ensure sufficient power the data analyses were limited to three years. The overall proportion achieving suppression of HBV replication was 57.4%, 79.0% and 85.6% at one, two and three years, respectively. No effect of prior or concomitant 3TC/FTC was shown. Virological rebound on TDF treatment was rare.

**Interpretation:**

TDF suppresses HBV to undetectable levels in the majority of HBV/HIV coinfected patients with the proportion fully suppressed continuing to increase during continuous treatment. Prior treatment with 3TC/FTC does not compromise efficacy of TDF treatment. The use of combination treatment with 3TC/FTC offers no significant benefit over TDF alone.

## Introduction

Approximately 10% of people infected with HIV are coinfected with hepatitis B virus (HBV). Among populations with access to antiretroviral therapy (ART), in whom serious opportunistic infections have become a rare event, liver diseases including HBV infection represent a major cause of morbidity and mortality. [1] Since the life-cycles of HIV and HBV both utilise a reverse transcriptase enzyme, some drugs that inhibit reverse transcriptase have activity against both viruses. Guidelines now recommend tenofovir (TDF) in combination with lamivudine (3TC) or emtricitabine (FTC) as first-line therapy for patients with HIV/HBV coinfection. [2] Many studies have reported on the effect of TDF, either with or without 3TC or FTC, in treatment-naïve or experienced patients, however many studies are small and with relatively short follow-up.

It is uncertain what proportion of patients achieves suppression of HBV DNA (viral load) and whether those in whom suppression is not seen after one year may achieve HBV suppression later. It is also unclear to what extent, if at all, those with complete suppression may relapse despite continued treatment, e.g. in case of development of resistance mutations. Finally, it remains uncertain whether sequential treatment, for example with 3TC initially and TDF later, compromises the chance of successful treatment with TDF.

A recent meta-analysis examined all randomised controlled trials of treatment for HBV but excluded patients with HIV coinfection and only compared responses at 12 months. [3] Outcomes included both virological (undetectable HBV viral load – excluded if the lower limit of detection was greater than 1000 copies/mL) and biochemical responses, HBeAg loss or seroconversion to anti-HBe, HBsAg loss, histological improvement and serious adverse events.

We carried out this meta-analysis of data from patients coinfected with HIV to amalgamate all available evidence and to answer the following questions:

- what proportion of patients achieve HBV viral load suppression on TDF?
- does the rate of suppression differ in those with prior 3TC experience?
- does the rate of suppression differ in those treated with combination therapy compared with TDF monotherapy?
- how common is HBV rebound on TDF?

## Methods

The systematic review was carried out following the guidance laid out in the PRISMA statement [4].

### Search Strategy and Selection Criteria

Studies included were those that described HBV/HIV coinfected individuals treated with TDF with or without 3TC and/or FTC for a period of at least one year and that gave results of quantification of plasma HBV viral load at yearly intervals (at a minimum) while on TDF treatment. Studies included could be randomised controlled trials or prospective or retrospective cohort studies. Patients with undetectable plasma HBV viral load at baseline were excluded since their inclusion gives a falsely high estimate of the effect of treatment. Baseline HBV viral load data was not given for 20 patients in three studies (see Table 1). The analysis was restricted to patients on TDF treatment, with or without 3TC and/or FTC. In this analysis inclusion bias could be considerable if patients who failed to suppress either stopped taking TDF or had progressive liver disease and so dropped out. This would leave a higher proportion of patients with a good response, overestimating the treatment effect. However very little data required to deal with this has been published. Further analysis of individual patient data was carried out where this was available or was provided in the process of performing the current analysis (Table 1).

**Table 1:** Characteristics of included studies.

Author	Publication year	Study design	N included in meta-analysis	HBeAgpositive	Baseline HBV viral load test	Level of detection^a^ IU/mL	Duration of follow-up	Additional data	Funding
Avihingsanon [5]	2010	RCT	10	6/10	Yes	34	48 weeks	No	GS
Bani-Sadr [6]	2004	Prospective cohort	6	3/6	Yes	40	96 weeks	Yes c	NS
Butt [7]	2006	Retrospective cohort	5	Unknown	Yes	20	36 months	No	d
de Vries-Sluijs [8]	2010	Prospective cohort	78	67/82	Yes	20	10–84 months	Yes c	GS
Dore [9]	2004	RCT	5	4/5	Yes	200	48 weeks	No	GS, e
Engell [10]	2011	Retrospective cohort	24	18/31	Yes	6	24 months	No	f
Gutiérrez [11]	2008	Retrospective cohort	6	Unknown	Yes	Not given	15–45 months	No c	NS
Jain [12]	2007	Retrospective cohort	28	27/28	Yes	400	12–24 months	Yes	NS
Kosi [13]	2012	Retrospective cohort	49	35/49	Yes	20	2–171 months	Yes c	NS
Kuzushita [14]	2010	Prospective cohort	16	15/16	Yes	60	6–63 months	Yes c	NS
Lee [15]	2009	Retrospective cohort	17	34/43	7/17	b 100–200	12–63 months	Yes c	g
Marcelin [16]	2003	Retrospective cohort	10	9/10	Yes	40	12 months	No	NS
Matthews [17]	2008	RCT	22	13/22	Yes	34	48 weeks	No	GS
Nelson [18]	2006	RCT	39	Unknown	Yes	80	48 weeks	No	NS
Nüesch [19]	2008	RCT	5	2/5	Yes	400	48–96 weeks	Yes c	R, h
Peters [20]	2006	RCT	18	23/27	Yes	40	48 weeks	Yes	i
Quiros-Roldan [21]	2008	Retrospective cohort	10	5/10	Yes	400	63–258 weeks	No c	j
Rodriguez [22]	2010	Prospective cohort	6	6/6	Yes	25	48 weeks	No	GSK
Schmutz [23]	2006	Prospective cohort	75	75/75	Yes	200	26–206 weeks	Yes	NS
Stephan [24]	2005	Retrospective cohort	23	19/31	Yes	400	48 weeks	Yes	NS
Tan [25]	2009	Retrospective cohort	39	39/39	38/39	b 100–2,000	69–290 weeks	Yes	None
Tuma [26]	2008	Retrospective cohort	38	Unknown	29/38	10	48 weeks	No	NS
van Bommel [27]	2004	Prospective cohort	21	21/21	Yes	80	72–130 weeks	No	k

RCT: Randomised controlled trial. GS: Gilead Sciences. R: Roche. GSK: GlaxoSmithKline. NS: not stated.

a Copies/mL converted to IU/mL by dividing by 5.

b The limit of detection of the HBV viral load assays used fell during the course of follow up in two studies.

c Individual patient data available.

d National Institutes of Health/National Institute on Drug Abuse.

e Commonwealth Department of Health and Ageing (Canberra, Australia).

f supported in part by National Institute of Allergy and Infectious Diseases.

g Medical Student Summer Research Training Program, supported through grants from the National Institutes of Health; Wake Forest University School of Medicine Departments, Centers, and Institutes; and private gifts.

h Swiss National Science Foundation through the Swiss HIV Cohort Study, the Wilsdorf, Sidaide, and de Brocard Foundations, Geneva, from the Departments of Social Affairs and Economics, Geneva.

i In part by the Adult AIDS Clinical Trials Group (ACTG) funded by the National Institute of Allergy and Infectious Diseases; virology support funding by the NIH/NIAID and the Adult ACTG Central Group; the Birmingham VA Medical Center, UAB CFAR core clinic and laboratory facilities; and NIDDK UCSF Liver Center.

j Italian Ministry of University.

k In part by the German BMBF Network of Competence for Viral Hepatitis (Hep Net).

TDF received approval for the treatment of HIV infection from the United States Food and Drug Administration (FDA) in October 2001 and from the European Medicines Agency in February 2002. (FDA approval for the treatment of chronic HBV infection was granted in August 2008.) The first reports of the use of TDF in treating HBV infection were presented in 2002. Web of Science, Embase and Medline were searched, including all years. Conference abstracts from The Liver Meeting (American Association for the Study of Liver Diseases), The International Liver Congress (European Association for the Study of the Liver) and the Conference on Retroviruses and Opportunistic Infections were searched for the years 2002–2010.

To search databases, a combination of key terms was used including “hepatitis”, “HIV”, and “tenofovir”, limited to articles with human subjects and written in English (Appendix S1). Conference abstracts were searched online or by hand. Other publications that were discovered from the reference lists in publications reviewed were also included.

### Data Collection

Studies were screened initially by title and then data was collected by HP from the full article of all published studies and from conference posters, or conference abstracts if posters were not available. Some studies met the eligibility criteria except that the published report did not include data on the number with undetectable HBV viral load at one year, or information on prior or concomitant drug exposure. The authors of these studies were contacted by email and asked to provide additional data if available. Additional, unpublished data was obtained from the authors of 11 of the 23 sources included (Table 1). The authors of one conference report provided an article that superseded the conference report and which had been accepted and published online but that had not been discovered in the search [13].

Data collected consisted of type of study, source of study funding, number of HBV/HIV coinfected participants, number HBeAg positive at study entry, prior 3TC/FTC exposure, drug regimens used during study period, length of follow-up, type of HBV viral load test used and lower limit of detection, numbers tested for HBV viral load at yearly intervals, and numbers with undetectable HBV viral load at yearly intervals. To maximise power and in the absence of any evidence suggesting a difference in effect on HBV between 3TC and FTC, exposure to these two were grouped together.

Results were stratified by treatment into four groups. Group A consisted of patients who had no prior exposure to 3TC/FTC and who were treated with TDF without concomitant 3TC/FTC, Group B those without prior exposure to 3TC/FTC treated with TDF in combination with 3TC/FTC, Group C those with prior exposure to 3TC/FTC but treated with TDF without 3TC/FTC, and Group D those with prior exposure to 3TC/FTC treated with TDF in combination with 3TC/FTC.

### Statistical Analysis

Statistical analysis was carried out using STATA version 10.1. The main outcome measure used was the proportion of patients tested who had a HBV viral load below the limit of detection at each of any available yearly time intervals. 95% confidence intervals for these proportions were calculated for each time point in each study and for the aggregate results.

To detect potential sources of bias, assay cut-off was plotted against proportion suppressed at one year. Publication bias was examined using funnel plots, in which asymmetry with a lack of poorly performing studies (to the left) would suggest such studies were not published.

Multilevel mixed effects logistic regression (XTMELOGIT command) was used to assess the effect of prior exposure to, and combination treatment with 3TC/FTC on the probability of viral suppression, with individual studies fitted as a random effect to account for clustering (Appendix S2). This implicitly weights each study by the amount of information it contains. Since there was no association of assay cut-off with rate of suppression the model was not adjusted for cut-off. The significance of between study heterogeneity was assessed by a likelihood-ratio test comparing the mixed effects model with a standard logistic regression model.

Models were re-run with an interaction term to examine whether the effect of concomitant 3TC/FTC was the same in both those naïve and those exposed to prior 3TC/FTC. As sensitivity analyses, the model was re-run (i) including only larger studies (reporting at least ten patients), (ii) including all studies apart from one that appeared as an outlier on the funnel plot. [8] and (iii) with a term for study design.

All authors had access to the data in the study and reviewed and approved the manuscript.

## Results

**Figure 1:**
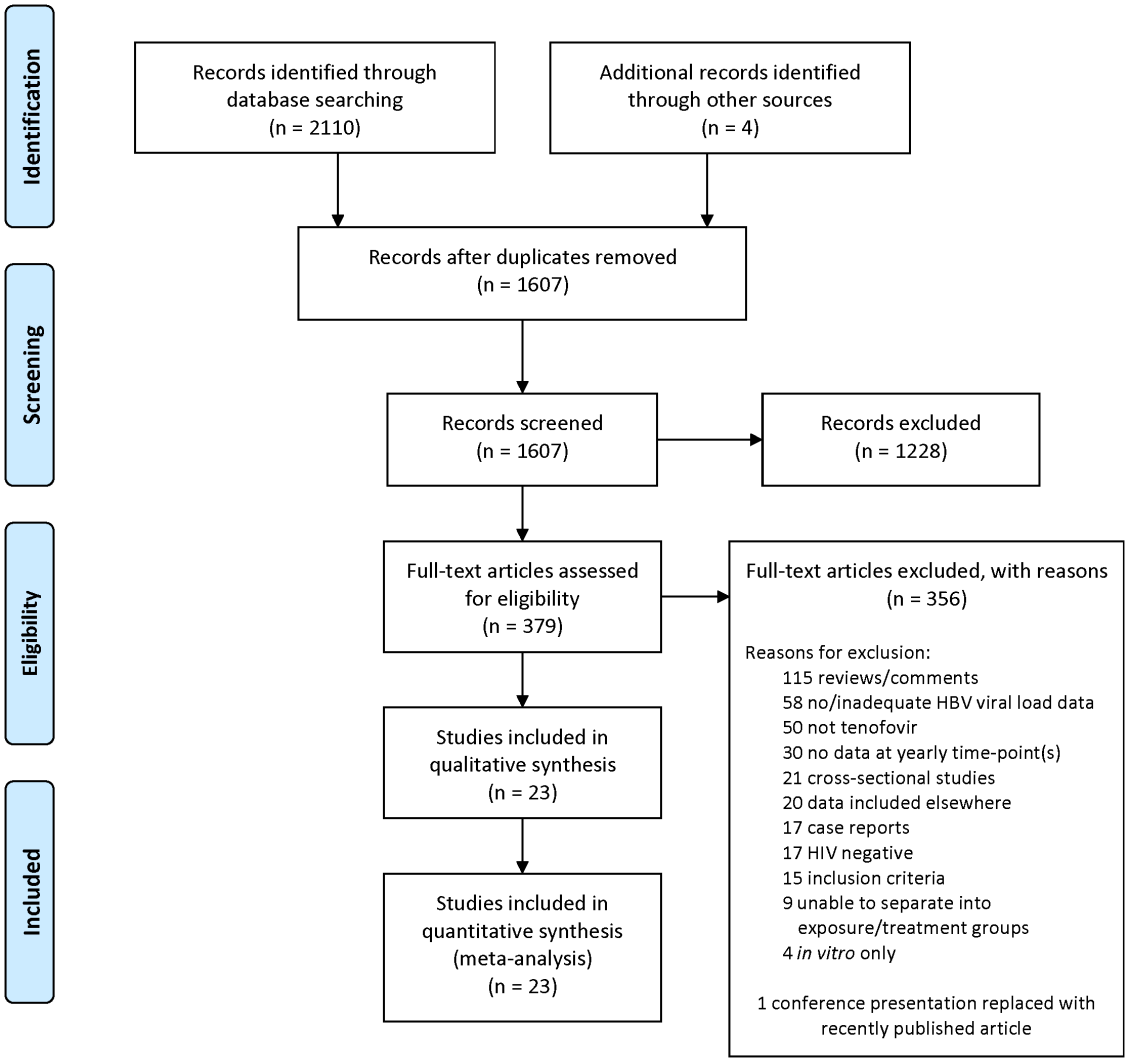
Summary of study search and inclusion (PRISMA flow diagram).

The initial searches produced 2,110 references which, after duplicates were removed, referred to 1,607 publications. Publications were then screened by title and if necessary by abstract to remove those clearly not meeting the eligibility criteria. This left 379 published articles. The full text of articles and posters was then checked for eligibility (or abstracts if the full article or poster was not available). 356 were removed as ineligible (as described in Figure 1) and 23 included in the analysis.

**Figure 2:**
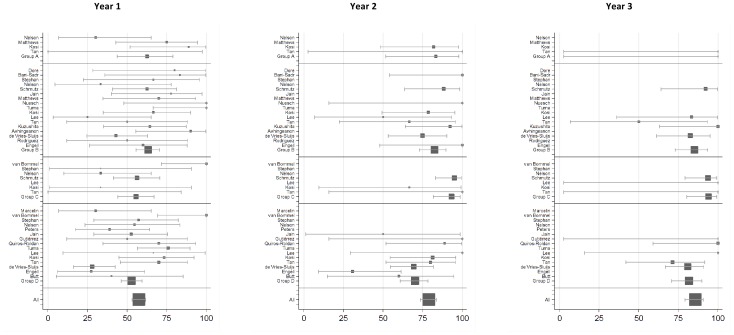
Forest plots of study arms included in the meta-analysis at years 1–3.

Study characteristics are given in Table 1. Although data was included from six randomised controlled trials, allocation of TDF vs. TDF plus 3TC was randomised in only two. [17], [18] Some studies included patients in more than one treatment group (for example both patients with and without prior exposure to 3TC), giving 43 study arms in total (Table 2 and Figure 2).

**Table 2:** Results available for meta-analysis.

	Year	1	2	3	4	5	6	7
Group	Author	S/N	S/N	S/N	S/N	S/N	S/N	S/N
A	Nelson	3/10						
	Matthews	9/12						
	Kosi	8/9	9/11					
	Tan	0/1	1/1	1/1				
B	Dore	4/5						
	Bani-Sadr	5/6	6/6					
	Stephan	4/6						
	Nelson	2/6						
	Schmutz	15/24	15/17	12/13	4/5			
	Jain	7/9						
	Matthews	7/10						
	Nüesch	5/5	2/2					
	Tuma	9/9						
	Kosi	8/12	11/14					
	Lee	2/8	2/4	5/6	2/2	1/1		
	Tan	3/6	4/6	2/4	4/4	3/3		
	Kuzushita	9/14	12/13	8/8	5/5	5/5		
	Avihingsanon	9/10						
	de Vries-Sluijs	12/28	18/24	19/23	14/14	6/6	1/1	
	Rodriguez	3/6						
	Engell	6/10	5/5					
C	van Bommel	11/11						
	Stephan	1/3						
	Nelson	4/12						
	Schmutz	27/48	38/40	30/32	9/9			
	Lee			1/1				
	Kosi	1/3	2/3					
	Tan	0/2	2/2	1/1				
D	Marcelin	3/10						
	van Bommel	10/10						
	Stephan	8/14						
	Nelson	6/11						
	Peters	7/18						
	Jain	10/19	1/2					
	Gutiérrez	3/6	2/2	1/1				
	Quiros-Roldan	7/10	8/9	7/7	5/5	1/1		
	Tuma	22/29						
	Lee	2/3	3/3	2/2				
	Kosi	11/15	13/16					
	Tan	14/20	12/15	10/14	7/8	7/9		
	de Vries-Sluijs	14/50	34/49	38/47	33/38	21/23	8/8	1/1
	Engell	3/11	4/13					
	Butt	2/5	3/5	3/5				

S: number of HBV viral load test results showing viral suppression (below the level of detection).

N: number of patients with a HBV viral load test performed.

In cases where insufficient data was published to categorise participants for this meta-analysis (for example if it was impossible to separate according to prior/concomitant treatment or if individuals with undetectable HBV viral load at baseline were included [29]), authors were contacted for further information. Those studies for which published data has been augmented by additional information are so labelled in Table 1.

**Figure 3:**
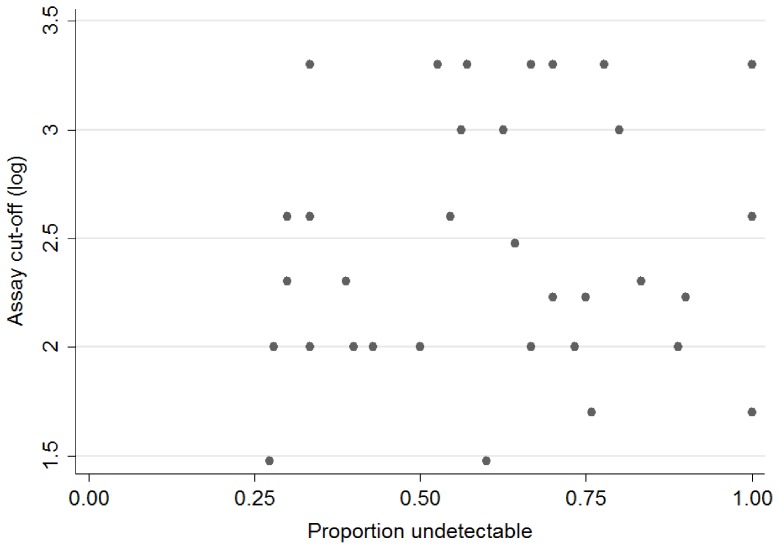
Log of HBV viral load assay cut-off against proportion undetectable at one year.

Studies used assays with widely varying cut-offs for the detection of HBV (Table 1). This could have introduced bias, with the use of more sensitive assays resulting in an apparent lower rate of suppression. However plotting the proportion undetectable against the logarithm of the cut-off value showed no clear pattern (Figure 3) and the cut-off was ignored in further analyses.

The overall proportion suppressed was 57.4% (95% CI: 53.0–61.7%), 79.0% (95% CI: 73.6–83.8%), and 85.6% (95% CI: 79.2–90.7%) after one, two, and three years of treatment with TDF (Table 3).

**Table 3:** Suppression at yearly time points.

	Number suppressed/number tested (% suppressed)									
	Group A		Group B		Group C		Group D		All	
Year	S/N	%	S/N	%	S/N	%	S/N	%	S/N	%
1	20/32	62.5	110/174	63.2	44/79	55.7	122/231	52.8	296/516	57.4
2	10/12	83.3	75/91	82.4	42/45	93.3	80/114	70.2	207/262	79.0
3	1/1	100	46/54	85.2	32/34	94.1	58/71	81.7	137/160	85.6

S: number of HBV viral load test results showing viral suppression (below the level of detection).

N: number of patients with a HBV viral load test performed.

**Figure 4:**
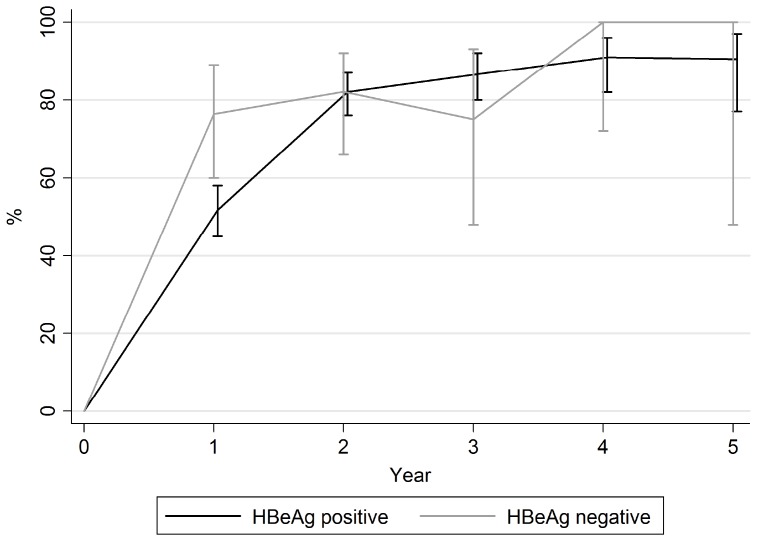
Percentage with undetectable HBV viral load over time, by HBeAg status.

It was possible to assess rates of virological suppression by HBeAg status for patients from ten of the included studies. [6], [8], [13], [14], [19], [21]–[23], [25], [27] For HBeAg positive and negative patients respectively the proportion fully suppressed was 51.8%, 82.0%, 86.6% and 76.3%, 82.1%, 75.0% at one, two and three years (Figure 4). After one year of treatment, a higher proportion of HBeAg negative than HBeAg positive individuals had a fully suppressed HBV viral load (p = 0.005). However, after one year the rates of suppression were not significantly different.

Table 4 shows the effects of prior and concomitant 3TC/FTC on virological suppression. Effects are given for all patients and also stratified by prior or concomitant treatment with 3TC/FTC as appropriate. Overall, at one year prior exposure to 3TC had an odds ratio of 0.69 (95% CI: 0.45 to 1.08) and treatment with 3TC/FTC in addition to TDF of 1.24 (95% CI: 0.68 to 2.24), neither being statistically significant. The effect of prior exposure to 3TC/FTC was similar, but also not statistically significant, at each of one, two, and three years. The effect of concomitant treatment with 3TC/FTC favoured dual therapy at one year but TDF monotherapy at years two and three, but these effects were again not statistically significant. The odds ratios in the stratified analyses were similar to the effects overall but with even wider confidence intervals. There was no evidence of an interaction between prior and concomitant 3TC/FTC treatment (p = 0.98 at 1 year, p = 0.14 at 2 years and p = 0.99 at 3 years). Between-study heterogeneity, allowing for the effects of prior and concomitant 3TC/FTC treatment, was significant (p<0.01) at year 1 but not at year 2 (p = 0.48) or at year 3 (p = 1.0).

**Table 4:** Multivariate logistic regression analysis of effects of prior and concomitant 3TC/FTC on virological suppression.

	Effect of prior 3TC/FTC						Effect of concomitant 3TC/FTC					
	Monotherapy		Dual therapy		Overall		3TC/FTC naive		Prior 3TC/FTC exposure		Overall	
Year	OR	95% CI	OR	95% CI	OR	95% CI	OR	95% CI	OR	95% CI	OR	95% CI
1	0.37	0.09 to 1.59	0.64	0.39 to 1.06	0.69	0.45 to 1.08	1.13	0.40 to 3.15	2.14	0.75 to 6.12	1.24	0.68 to 2.24
2	0.80	0.06 to 11.50	0.55	0.20 to 1.49	0.69	0.35 to 1.39	0.94	0.19 to 4.70	0.23	0.03 to 1.64	0.37	0.11 to 1.30
3	–	–	0.77	0.30 to 2.03	0.75	0.29 to 1.96	–	–	0.28	0.06 to 1.96	0.25	0.05 to 1.14

Monotherapy: patients treated with TDF without concomitant 3TC/FTC, i.e. groups A and C.

Dual therapy: patients treated with TDF with concomitant 3TC/FTC, i.e. groups B and D.

3TC/FTC naïve: patients not previously exposed to 3TC/FTC before TDF treatment, i.e. groups A and B.

Prior 3TC/FTC exposure: patients previously exposed to 3TC/FTC before TDF treatment, i.e. groups C and D.

OR: odds ratio.

CI: confidence interval The effects comparing groups A and C and comparing groups A and B in year 3 were non-estimable as there is only one patient in group A.

The proportion suppressed increased over time and reached 100% overall (Table 2). The number of patients in follow-up at each year declined, however of the 379 patients in studies with more than one year of follow-up, individual patient data was available for 187 (49.3%) and in these patients dropping out (i.e. no later HBV viral load test result being available) was more likely at every time point for patients with suppressed HBV than for those with detectable HBV (non-significant – data not shown).

Virological rebound on TDF was rare, with no cases seen in 16 of 23 studies. Three studies reported a single patient with an increase in HBV viral load on TDF treatment, [17], [21], [25] three had two patients, [7], [14], [22] and one had three [8] though in three of these studies the size of the increases were not reported, in two the increases were very small (0.1 to 0.3 log), and only two had patients with an increase of at least one log (one in each study). [7], [22] Unfortunately no discussion of these two cases was given, in particular there were no data on drug compliance and treatment adherence.

**Figure 5:**
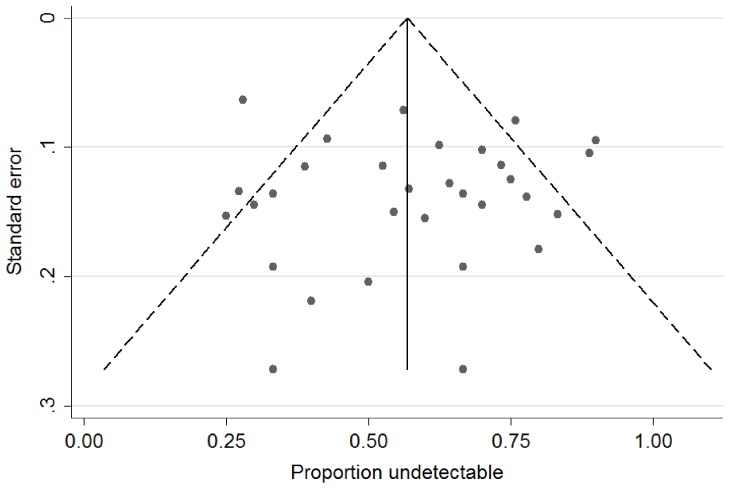
Funnel plot of standard error against proportion undetectable at one year – all study arms (with pseudo 95% confidence limits).

The funnel plot (Figure 5) shows the standard error against the proportion undetectable at one year, with the vertical line marking the summary estimate of the treatment effect (derived using fixed-effect meta-analysis). [28] The plot is symmetrical with no suggestion of publication bias. There is considerable heterogeneity in the effect found in larger studies (appearing higher up on the graph with a lower standard error), with one apparent outlier with a low proportion undetectable despite large size (de Vries-Sluijs, [8] Group D). Separate funnel plots of each arm in the analysis also show no publication bias (not shown). Repeating the regression analysis after excluding the outlier study arm and after excluding small studies (with less than ten patients) made no significant difference to the results. The model included a term for study design and showed that study design had no significant impact on the results, with p values of 0.76, 0.54 and 0.42 at 1, 2 and 3 years in the overall analysis.

## Discussion

This review of HBV/HIV coinfected patients treated with TDF results demonstrates lasting virological suppression of HBV replication to below the level of detection, with the proportion suppressed increasing to 100% over time, though with small numbers at later time points. Few patients experience virological failure on treatment.

However several reservations should be noted. Firstly most of the studies included were observational in design and patients dropping out were not well characterised. Secondly, in this meta-analysis we compare different treatment groups though allocation to these was randomised in only two studies. [17], [18] Thirdly the numbers of patients included in the meta-analysis declines rapidly over time.

The proportion with undetectable HBV at one year (59%) was lower than the proportion found in HIV negative patients receiving TDF for treatment of HBV infection. For example, a multicentre cohort study found that, of 54 HIV-negative patients treated with TDF and FTC, 60% of whom were HBeAg positive, the probability of attaining an undetectable HBV viral load was 76% at one year and 94% at two years. [30] Similarly, in a large randomised controlled trial comparing TDF with adefovir, Marcellin found 93% of 250 HBeAg negative and 76% of 176 HBeAg positive patients randomised to TDF had an undetectable viral load (<400 copies/mL) at 48 weeks (97% and 83% respectively of those still on TDF at 48 weeks) [31].

In the latter study, ten patients (2.3%) had virological breakthrough (defined in that study as detectable HBV after an undetectable result or an increase in HBV viral load by a factor of ten from nadir). [31] Of the 550 patients in the current study, we identified 12 (2.4%) with a rise in HBV viral load on TDF treatment (although at least five of these 12 had less than a one log rise from nadir) which is comparable to HBV-monoinfected patients. However other published data in coinfected patients have found far higher rates, for example 9 (17%) of 52 patients followed up for a median of 34 months in one retrospective cohort study (which was not included in the current meta-analysis as data on HBV viral load suppression was only given at the end of follow-up and not at yearly time points) [32].

The high rate of virological suppression and low rate of breakthrough may be related to the low chance of developing TDF-resistance mutations. In HBV/HIV coinfected patients treated with lamivudine as the only drug active against HBV, resistance develops in about 90% after four years [33] whereas mutations associated with TDF resistance, such as the combination of rtL180M, rtM204V/I and rtA194T [34] or N236T with A181V, [35] have only rarely been seen and are of uncertain significance [36]–[38].

No statistically significant effect of prior 3TC/FTC exposure or of concomitant 3TC/FTC use was found and thus no evidence to support the hypotheses that prior exposure may make subsequent treatment less effective or that concomitant use of 3TC/FTC may give a higher rate of suppression. However given the modest number of patients available for inclusion in the meta-analysis, the confidence intervals were wide and we could not exclude the possibility of moderately strong effects in either direction. In HIV-negative patients TDF monotherapy is as effective for HBV as combination therapy with TDF and 3TC/FTC with suppression rates (<400 copies/mL) of 81% at one year in both arms of an RCT using TDF alone or TDF/FTC combination therapy, and 88% and 85% respectively at three years [39], [40].

The main concern with sequential treatments that fail to fully suppress the viral load is that resistance may develop and that cross-resistance could reduce the efficacy of subsequent drugs. TDF resistance is yet to be clearly demonstrated but it may be that the risk of cross-resistance is higher with drugs that are more similar to TDF in structure than 3TC/FTC. However HBV mono-infected patients failing to achieve virologic suppression with adefovir have also been shown to respond well to TDF [41]–[43].

A second mechanism by which prior treatment exposure could reduce the apparent effectiveness of subsequent TDF is through introducing bias, in that patients failing one regimen for reasons other than lack of potency (such as poor adherence to therapy) may go on to fail other regimens but again, no such reduction in the effect of TDF in those with prior exposure to 3TC/FTC was found and so the effect of any such bias must be small.

As stated above, TDF received FDA approval in late 2001 and thus clinical experience to date is limited to just over one decade. Although this review includes data to a maximum of seven years, a lack of data limited the main regression analyses to three years. Patients with HIV require lifelong treatment and patients with HBV coinfection are likely to require the same. The possibility of safe discontinuation of HBV treatment may be limited to patients who clear serum hepatitis B surface antigen (HBsAg). However the probability of HBsAg loss is low with a rate of approximately 2.5% per year [44], [45] with the predicted median time to HBsAg seroclearance in HBeAg positive patients treated with TDF being 18 years (IQR 10–28 years) [46].

A limitation of this study is that it does not include analysis of the adverse effects of treatment. Future studies with longer follow-up duration will be required to determine the risk of treatment associated adverse effects, such as renal and bone toxicity, in patients exposed to TDF for many decades.

In conclusion, this meta-analysis shows that tenofovir suppresses HBV to undetectable levels in the majority of HBV/HIV coinfected patients, with the proportion fully suppressed increasing with time on treatment and with little if any virological rebound on treatment. Prior treatment with 3TC/FTC does not alter the efficacy of TDF treatment. Combination treatment with 3TC/FTC offers no significant benefit over tenofovir alone.

## Supporting Information

Appendix S1 **Literature search strings.**
(DOC)

Appendix S2 **Stata code.**
(DOC)

## References

[1] Weber R, Sabin CA, Friis-Moller N, Reiss P, El-Sadr WM, et al (2006) Liver-related deaths in persons infected with the human immunodeficiency virus: the D:A:D study. Arch Intern Med 166: 1632–1641. 16908797 10.1001/archinte.166.15.1632

[2] Brook G, Main J, Nelson M, Bhagani S, Wilkins E, et al (2010) British HIV Association guidelines for the management of coinfection with HIV-1 and hepatitis B or C virus 2010. HIV Med 11: 1–30. 10.1111/j.1468-1293.2009.00781.x 20059574

[3] Woo G, Tomlinson G, Nishikawa Y, Kowgier M, Sherman M, et al (2010) Tenofovir and entecavir are the most effective antiviral agents for chronic hepatitis B: a systematic review and Bayesian meta-analyses. Gastroenterology 139: 1218–1229. 20600036 10.1053/j.gastro.2010.06.042

[4] Moher D, Liberati A, Tetzlaff J, Altman DG (2009) Preferred reporting items for systematic reviews and meta-analyses: the PRISMA statement. PLoS Med 6: e1000097. 19621072 10.1371/journal.pmed.1000097 PMC2707599

[5] Avihingsanon A, Lewin SR, Kerr S, Chang JJ, Piyawat K, et al (2010) Efficacy of tenofovir disoproxil fumarate/emtricitabine compared with emtricitabine alone in antiretroviral-naive HIV-HBV coinfection in Thailand. Antivir Ther 15: 917–922. 20834105 10.3851/IMP1645

[6] Bani-Sadr F, Palmer P, Scieux C, Molina JM (2004) Ninety-six-week efficacy of combination therapy with lamivudine and tenofovir in patients coinfected with HIV-1 and wild-type hepatitis B virus. Clin Infect Dis 39: 1062–1064. 15472862 10.1086/424012

[7] Butt AA (2006) Tenofovir for chronic hepatitis B virus infection in HIV-coinfected patients. AIDS Reader 16: 219–222. 16617931

[8] de Vries-Sluijs TE, Reijnders JG, Hansen BE, Zaaijer HL, Prins JM, et al (2010) Long-term Therapy With Tenofovir Is Effective for Patients Co-Infected With Human Immunodeficiency Virus and Hepatitis B Virus. Gastroenterology 139: 1934–1941. 20801123 10.1053/j.gastro.2010.08.045

[9] Dore GJ, Cooper DA, Pozniak AL, DeJesus E, Zhong L, et al (2004) Efficacy of tenofovir disoproxil fumarate in antiretroviral therapy-naive and -experienced patients coinfected with HIV-1 and hepatitis B virus. J Infect Dis 189: 1185–1192. 15031786 10.1086/380398

[10] Engell CA, Pham VP, Holzman RS, Aberg JA (2011) Virologic Outcome of Using Tenofovir/Emtricitabine to Treat Hepatitis B in HIV-Coinfected Patients. ISRN Gastroenterol 2011: 405390. 21991507 10.5402/2011/405390 PMC3168392

[11] Gutierrez S, Guillemi S, Jahnke N, Montessori V, Harrigan PR, et al (2008) Tenofovir-based rescue therapy for advanced liver disease in 6 patients coinfected with HIV and hepatitis B virus and receiving lamivudine. Clin Infect Dis 46: e28–30. 18181733 10.1086/525857

[12] Jain MK, Comanor L, White C, Kipnis P, Elkin C, et al (2007) Treatment of hepatitis B with lamivudine and tenofovir in HIV/HBV-coinfected patients: factors associated with response. J Viral Hepat 14: 176–182. 17305883 10.1111/j.1365-2893.2006.00797.x

[13] Kosi L, Reiberger T, Payer BA, Grabmeier-Pfistershammer K, Strassl R, et al (2012) Five-year on-treatment efficacy of lamivudine-, tenofovir- and tenofovir+emtricitabine-based HAART in HBV–HIV-coinfected patients. J Viral Hepat 19: 801–10. 23043387 10.1111/j.1365-2893.2012.01601.x

[14] Kuzushita N, Suemura S, Toyama T, Hasegawa H, Yuguchi K, et al.. (2010) Long-term efficacy of lamivudine/emtricitabine and tenofovir combination therapy on HBV/HIV co-infected Japanese patients. 61st Annual Meeting of the American Association for the Study of Liver Diseases. Boston, U.S.A.

[15] Lee T, Nunez M (2009) Longer duration of HBV-active antiretroviral Therapy is linked to favorable virological outcome in HIV-HBV co-infected patients. HIV Clinical Trials 10: 153–159. 19632954 10.1310/hct1003-153

[16] Marcelin AG, Tubiana R, Benhamou Y, Katlama C, Calvez V, et al.. (2003) Long-term tenofovir treatment of lamivudine-resistant chronic hepatitis B in HIV co-infected patients. 10th Conference on Retroviruses and Opportunistic Infections. Boston, U.S.A.

[17] Matthews GV, Avihingsanon A, Lewin SR, Amin J, Rerknimitr R, et al (2008) A randomized trial of combination hepatitis B therapy in HIV/HBV coinfected antiretroviral naive individuals in Thailand. Hepatology 48: 1062–1069. 18697216 10.1002/hep.22462

[18] Nelson M, Bhagani S, Fisher M, Leen C, Brook G, et al.. (2006) A 48-week Study of Tenofovir or Lamivudine or a Combination of Tenofovir and Lamivudine for the Treatment of Chronic Hepatitis B in HIV/HBV-co-infected Individuals. 13th Conference on Retroviruses and Opportunistic Infections. Denver, U.S.A.

[19] Nüesch R, Ananworanich J, Srasuebkul P, Chetchotisakd P, Prasithsirikul W, et al (2008) Interruptions of tenofovir/emtricitabine-based antiretroviral therapy in patients with HIV/hepatitis B virus co-infection. AIDS 22: 152–154. 18090405 10.1097/QAD.0b013e3282f303bf

[20] Peters MG, Andersen J, Lynch P, Liu T, Alston-Smith B, et al (2006) Randomized controlled study of tenofovir and adefovir in chronic hepatitis B virus and HIV infection: ACTG A5127. Hepatology 44: 1110–1116. 17058225 10.1002/hep.21388 PMC4114764

[21] Quiros-Roldan E, Calabresi A, Lapadula G, Tirelli V, Costarelli S, et al (2008) Evidence of long-term suppression of hepatitis B virus DNA by tenofovir as rescue treatment in patients coinfected by HIV. Antivir Ther 13: 341–348. 18572746

[22] Rodriguez AE, Dejesus E, Williams V, Irlbeck D, Ross L, et al (2010) Efficacy and Safety of Abacavir/Lamivudine/Zidovudine Plus Tenofovir in HBV/HIV-1 Coinfected Adults: 48-Week Data. Open AIDS J 4: 167–170. 21253458 10.2174/1874613601004010167 PMC3023942

[23] Schmutz G, Nelson M, Lutz T, Sheldon J, Bruno R, et al (2006) Combination of tenofovir and lamivudine versus tenofovir after lamivudine failure for therapy of hepatitis B in HIV-coinfection. AIDS 20: 1951–1954. 16988516 10.1097/01.aids.0000247116.89455.5d

[24] Stephan C, Berger A, Carlebach A, Lutz T, Bickel M, et al (2005) Impact of tenofovir-containing antiretroviral therapy on chronic hepatitis B in a cohort co-infected with human immunodeficiency virus. J Antimicrob Chemother 56: 1087–1093. 16269552 10.1093/jac/dki396

[25] Tan LK, Gilleece Y, Mandalia S, Murungi A, Grover D, et al (2009) Reduced glomerular filtration rate but sustained virologic response in HIV/hepatitis B co-infected individuals on long-term tenofovir. J Viral Hepat 16: 471–478. 19457140 10.1111/j.1365-2893.2009.01084.x

[26] Tuma P, Bottecchia M, Sheldon J, Medrano J, Vispo E, et al.. (2008) Prior lamivudine (Lam) failure may delay time to complete HBV-DNA suppression in HIV patients treated with tenofovir plus Lam. 59th Annual Meeting of the American Association for the Study of Liver Diseases. San Francisco, U.S.A.

[27] van Bommel F, Wunsche T, Mauss S, Reinke P, Bergk A, et al (2004) Comparison of adefovir and tenofovir in the treatment of lamivudine-resistant hepatitis B virus infection. Hepatology 40: 1421–1425. 15565615 10.1002/hep.20464

[28] Sterne J, Harbord R (2004) Funnel plots in meta-analysis. The Stata Journal 4: 127–141.

[29] Lacombe K, Gozlan J, Boyd A, Boelle PY, Bonnard P, et al (2008) Comparison of the antiviral activity of adefovir and tenofovir on hepatitis B virus in HIV-HBV-coinfected patients. Antivir Ther 13: 705–713. 18771054 PMC2665195

[30] Si-Ahmed SN, Pradat P, Zoutendijk R, Buti M, Mallet V, et al (2011) Efficacy and tolerance of a combination of tenofovir disoproxil fumarate plus emtricitabine in patients with chronic hepatitis B: a European multicenter study. Antiviral Res 92: 90–95. 21767570 10.1016/j.antiviral.2011.07.003

[31] Marcellin P, Heathcote EJ, Buti M, Gane E, de Man RA, et al (2008) Tenofovir disoproxil fumarate versus adefovir dipivoxil for chronic hepatitis B. N Engl J Med. 359: 2442–2455. 10.1056/NEJMoa0802878 19052126

[32] Alvarez-Uria G, Ratcliffe L, Vilar J (2009) Long-term outcome of tenofovir disoproxil fumarate use against hepatitis B in an HIV-coinfected cohort. HIV Med 10: 269–273. 19210695 10.1111/j.1468-1293.2008.00683.x

[33] Benhamou Y, Bochet M, Thibault V, Di Martino V, Caumes E, et al (1999) Long-term incidence of hepatitis B virus resistance to lamivudine in human immunodeficiency virus-infected patients. Hepatology 30: 1302–1306. 10534354 10.1002/hep.510300525

[34] Sheldon J, Camino N, Rodes B, Bartholomeusz A, Kuiper M, et al (2005) Selection of hepatitis B virus polymerase mutations in HIV-coinfected patients treated with tenofovir. Antivir Ther 10: 727–734. 16218172

[35] Qi X, Xiong S, Yang H, Miller M, Delaney WEt (2007) In vitro susceptibility of adefovir-associated hepatitis B virus polymerase mutations to other antiviral agents. Antivir Ther 12: 355–362. 17591025

[36] Delaney WEt, Ray AS, Yang H, Qi X, Xiong S, et al (2006) Intracellular metabolism and in vitro activity of tenofovir against hepatitis B virus. Antimicrob Agents Chemother 50: 2471–2477. 16801428 10.1128/AAC.00138-06 PMC1489769

[37] Fung S, Mazzulli T, Sherman M, Popovic V (2009) Tenofovir (TDF) is effective in lamivudine (LAM)-resistant chronic hepatitis B patients who harbor rtA194T at baseline. 60th Annual Meeting of the American Association for the Study of Liver Diseases. Boston, U.S.A.

[38] Amini-Bavil-Olyaee S, Herbers U, Sheldon J, Luedde T, Trautwein C, et al (2009) The rtA194T polymerase mutation impacts viral replication and susceptibility to tenofovir in hepatitis B e antigen-positive and hepatitis B e antigen-negative hepatitis B virus strains. Hepatology 49: 1158–1165. 19263474 10.1002/hep.22790

[39] Berg T, Marcellin P, Zoulim F, Moller B, Trinh H, et al (2010) Tenofovir is effective alone or with emtricitabine in adefovir-treated patients with chronic-hepatitis B virus infection. Gastroenterology 139: 1207–1217. 20600025 10.1053/j.gastro.2010.06.053

[40] Berg T, Marcellin P, Moeller B, Trinh HN, Chan S, et al.. (2010) Tenofovir disoproxil fumarate (TDF) versus emtricitabine plus TDF (FTC/TDF) for treatment of chronic hepatitis B (CHB) in patients with persistent viral replication receiving adefovir dipivoxil: final week 168 results. 61st Annual Meeting of the American Association for the Study of Liver Diseases. Boston, U.S.A.

[41] Ong A, Wong VW, Wong GL, Chan HY, Tse CH, et al (2011) Management options for lamivudine-resistant chronic hepatitis B patients with suboptimal virological suppression by adefovir. Aliment Pharmacol Ther 34: 972–981. 21883327 10.1111/j.1365-2036.2011.04833.x

[42] Santos SA, Uriel AJ, Park JS, Lucas J, Carriero D, et al (2006) Effect of switching to tenofovir with emtricitabine in patients with chronic hepatitis B failing to respond to an adefovir-containing regimen. Eur J Gastroenterol Hepatol 18: 1247–1253. 17099372 10.1097/01.meg.0000243877.17444.5e

[43] Petersen J, Ratziu V, Buti M, Janssen HL, Brown A, et al (2012) Entecavir plus tenofovir combination as rescue therapy in pre-treated chronic hepatitis B patients: an international multicenter cohort study. J Hepatol 56: 520–526. 22037226 10.1016/j.jhep.2011.09.018

[44] Psevdos G Jr, Kim JH, Suh JS, Sharp VL (2010) Predictors of loss of hepatitis B surface antigen in HIV-infected patients. World J Gastroenterol 16: 1093–1096. 20205279 10.3748/wjg.v16.i9.1093 PMC2835785

[45] Martin-Carbonero L, Teixeira T, Poveda E, Plaza Z, Vispo E, et al (2010) Clinical and virological outcomes in HIV-infected patients with chronic hepatitis B on long-term nucleos(t)ide analogues. AIDS 25: 73–79. 10.1097/QAD.0b013e328340fde2 21076274

[46] Zoutendijk R, Zaaijer HL, de Vries-Sluijs TE, Reijnders JG, Mulder JW, et al (2012) Hepatitis B Surface Antigen Decline and Clearance During Long-Term Tenofovir Therapy in Patients Coinfected With HBV and HIV. J Infect Dis 15: 974–80. 10.1093/infdis/jis439 22782950

