# Supplementary material for: Suppression of HBV by Tenofovir in HBV/HIV Coinfected Patients: A Systematic Review and Meta-Analysis
Source: PLoS One. 2013 Jul 10;8(7):e68152. doi: 10.1371/journal.pone.0068152 (PMC3707972; doi:10.1371/journal.pone.0068152)
Supplement: Appendix S1 — Literature search strings. (DOC) [file pone.0068152.s001.doc]

ISI Web of Science

Topic=((TS=hepatitis OR TS=hbv) AND (TS=hiv OR TS=human immunodeficiency virus OR TS=AIDS OR TS=acquired immunodeficiency syndrome OR TS=acquired immune deficiency syndrome) AND (TS=tenofovir OR TS=TDF OR TS=truvada OR TS=viread))

Timespan=All Years. Databases=SCI-EXPANDED, SSCI, A&HCI, CPCI-S, CPCI-SSH.

EMBASE & MEDLINE

((exp HUMAN IMMUNODEFICIENCY VIRUS/) OR (hiv.ti,ab) OR (exp ACQUIRED IMMUNE DEFICIENCY SYNDROME/) OR (aids.ti,ab))

AND

((exp HEPATITIS/) OR (hbv.ti,ab) OR (hepatitis.ti,ab))

AND

((exp TENOFOVIR/) OR (exp TENOFOVIR DISOPROXIL/) OR (tenofovir.ti,ab) OR (tdf.ti,ab) OR

(viread.ti,ab) OR (truvada.ti,ab))

[Limit to: Human and English Language]
