# Supplementary material for: Suppression of HBV by Tenofovir in HBV/HIV Coinfected Patients: A Systematic Review and Meta-Analysis
Source: PLoS One. 2013 Jul 10;8(7):e68152. doi: 10.1371/journal.pone.0068152 (PMC3707972; doi:10.1371/journal.pone.0068152)
Supplement: Appendix S2 — Stata code. (DOC) [file pone.0068152.s002.doc]

The following Stata command produced the estimates in the “overall” columns in Table 4.

xi: xtmelogit u i.prior i.con i.studytype || study: , or

u 0 if not suppressed
1 if suppressed

prior 0 if not previously exposed to 3TC/FTC
1 if previously exposed to 3TC/FTC

con 0 if treated with TDF without concomitant 3TC/FTC
1 if treated with TDF with concomitant 3TC/FTC

studytype 1 if randomised controlled trial
2 if prospective cohort study
3 if retrospective cohort study

study numbers 1-23 for the 23 studies included (Error: Reference source not found)

Estimates of effects within strata were obtained by selecting appropriate cases e.g.

xi: xtmelogit u i.prior i.studytype if con==0 || study: , or
